# Dirammox Is Widely Distributed and Dependently Evolved in *Alcaligenes* and Is Important to Nitrogen Cycle

**DOI:** 10.3389/fmicb.2022.864053

**Published:** 2022-05-13

**Authors:** Ting-Ting Hou, Li-Li Miao, Ji-Sen Peng, Lan Ma, Qiang Huang, Ying Liu, Meng-Ru Wu, Guo-Min Ai, Shuang-Jiang Liu, Zhi-Pei Liu

**Affiliations:** ^1^State Key Laboratory of Microbial Resources, Institute of Microbiology, Chinese Academy of Sciences, Beijing, China; ^2^College of Life Sciences, University of Chinese Academy of Sciences, Beijing, China; ^3^State Key Laboratory of Microbial Technology, Shandong University, Qingdao, China

**Keywords:** nitrogen cycle, Dirammox, *Alcaligenes*, DnfA, environmental distribution

## Abstract

Nitrogen cycle is an essential process for environmental health. Dirammox (*dir*ect *amm*onia *ox*idation), encoded by the *dnfT1RT2ABCD* cluster, was a novel pathway for microbial N_2_ production defined in *Alcaligenes ammonioxydans* HO-1. Here, a copy of the cluster *dnfT1RT2ABCD* as a whole was proved to have existed and very conserved in all *Alcaligenes* genomes. Phylogenetic analyses based on 16S rRNA gene sequences and amino acid sequences of DnfAs, together with G + C content data, revealed that *dnf* cluster was evolved associated with the members of the genus *Alcaligenes*. Under 20% O_2_ conditions, 14 of 16 *Alcaligenes* strains showed Dirammox activity, which seemed likely taxon-related. However, the *in vitro* activities of DnfAs catalyzing the direct oxidation of hydroxylamine to N_2_ were not taxon-related but depended on the contents of Fe and Mn ions. The results indicated that DnfA is necessary but not sufficient for Dirammox activity. The fact that members of the genus *Alcaligenes* are widely distributed in various environments, including soil, water bodies (both freshwater and seawater), sediments, activated sludge, and animal–plant-associated environments, strongly suggests that Dirammox is important to the nitrogen cycle. In addition, *Alcaligenes* species are also commonly found in wastewater treatment plants, suggesting that they might be valuable resources for wastewater treatment.

## Introduction

The nitrogen cycle is important for nitrogen balance in the biosphere and global environmental health (Gruber and Galloway, 2008). Biological nitrogen removal of fixed nitrogen is not only an essential part of the natural nitrogen cycle (Gruber and Galloway, 2008), but also the basic principle in the development of nitrogen removal technologies in wastewater treatment (Kuypers et al., 2018). Up to date, only two pathways have been identified and recognized as biological N_2_ generation pathways, namely, nitrification/denitrification (Van Cleemput and Samater, 1995; Sigman and Fripiat, 2019) and anaerobic ammonia oxidation (anammox) (Mulder et al., 1995; Strous et al., 1999). In nitrification/denitrification pathway, ammonia oxidation (NH_4_^+^ → NH_2_OH → NO_2_^–^ → NO_3_^–^) is the energy source of autotrophic nitrifers, ammonia can be oxidized aerobically *via* hydroxylamine to nitrite, nitrate, or nitrogenous gas by a single-strain *Nitrospira* species (comammox, *com*plete *amm*onia *ox*idation), or by a combination of ammonia-oxidizing bacteria or ammonia-oxidizing archaea, and nitrite-oxidizing bacteria (Abell et al., 2010; Pester et al., 2012; Baptista et al., 2014). The enzyme ammonia monooxygenase catalyzes the oxidation of ammonia to hydroxylamine (Ensign et al., 1993; Gilch et al., 2009), which is then converted to NO by hydroxylamine oxidoreductase (Maalcke et al., 2014; Caranto and Lancaster, 2017). In denitrification pathway, nitrate or nitrite is sequentially reduced to N_2_ (NO_3_^–^ → NO_2_^–^ → NO → N_2_O → N_2_) when O_2_ is limited (Skiba, 2008). In anammox pathway, N_2_ is produced from oxidation of ammonia, with nitrite produced by nitrifiers as the electron acceptor (NH_4_^+^ + NO_2_^–^ → N_2_ or NH_4_^+^ + 1.32 NO_2_^–^ + 0.066 HCO_3_^–^ + 0.13 H^+^ → 1.02 N_2_ + 0.26 NO_3_^–^ + 0.066 CH_2_O_0_._5_N_0_._15_ + 2.03 H_2_O) under anaerobic conditions by anammox bacteria (phylum *Planctomycetes*) (Mulder et al., 1995; van de Graaf et al., 1996; Strous et al., 1999).

We previously isolated and characterized *Alcaligenes ammonioxydans* strain HO-1 (Wu et al., 2021) from a SHARON bioreactor treating ammonium-rich piggery wastewater (Du et al., 2016). Strain HO-1 could be tolerant to high ammonia load up to 3,400 mg/L and directly convert ammonia *via* hydroxylamine to N_2_ under aerobic conditions (Wu et al., 2021). The high ammonia tolerance property makes it a potential strain in wastewater treatment plants (WWTPs). Furthermore, it also demonstrated its ability to directly convert ammonia to N_2_ under aerobic conditions as a result of a novel pathway, termed Dirammox (*dir*ect *amm*onia *ox*idation) (Wu et al., 2021). It was also demonstrated that the production of N_2_ from ammonia in HO-1 was the activity of Dirammox, but not that of nitrite denitrification (Wu et al., 2021). This result was further confirmed in strain JQ135 (Xu et al., 2022), another *Alcaligenes* strain with the same ability of Dirammox as HO-1. The knockout of denitrification pathway in strain JQ135 did not change its N_2_ yield under aerobic conditions (Xu et al., 2022). This novel pathway is conducted by enzymes encoded by the gene cluster *dnfT1RT2ABCD* defined in the genome of HO-1; and just *dnfABC* could enable recombinant *Escherichia coli* strain to generate hydroxylamine and N_2_ from ammonia (Wu et al., 2021). These seven genes were annotated as 3-phosphoserine/phosphohydroxythreonine transaminase, PLP-dependent aminotransferase family protein, serine hydroxymethyltransferase, diiron *N*-oxygenase, 2Fe–2S iron–sulfur cluster binding domain-containing protein, glutamine amidotransferase, and pyridoxine/pyridoxal/pyridoxamine kinase, respectively (Wu et al., 2021). Among the seven genes, only *dnfA* encoded an *N*-oxygenase catalyzing the oxidation of hydroxylamine to N_2_, and its deletion resulted in the total loss of Dirammox activity in strain JQ135 (Xu et al., 2022). Therefore, *dnfA* was selected as a marker gene of Dirammox pathway for the following investigations.

Dirammox is quite different from the two known N_2_ production pathways mentioned above, namely, (1) N_2_ generation in Dirammox is quite efficient under physiological O_2_ levels by a single heterotrophic bacterium, while denitrification and anammox occur anaerobically; and (2) Dirammox is the simplest N loss process, of which N_2_ is directly generated from ammonia NH_3_^+^ (-NH_2_) *via* hydroxylamine by a single bacterium such as *A. ammonioxydans* HO-1. However, N_2_ produced by nitrification/denitrification is the result of the subsequent cooperation of multiple strains as mentioned above. Although an anammox bacterium could oxidize ammonia directly to N_2_ with nitrite as the final electron acceptor, it needs the cooperation of nitrifiers for providing nitrite (Kuypers et al., 2018).

Currently, there are five species in the genus, namely, *Alcaligenes faecalis*, *A. aquatilis* (Van Trappen et al., 2005), *A. pakistanensis* (Abbas et al., 2015), *A. endophyticus* (Lu et al., 2017), and *A. ammonioxydans* HO-1 (Wu et al., 2021). Members of the genus *Alcaligenes* are widely distributed in natural environments such as soil (Liu X. et al., 2016), water (Regar et al., 2016), as well as human and other vertebrates (Simmons et al., 1981; Kaliaperumal et al., 2006; Laham et al., 2017). With the disclosed Dirammox in *Alcaligenes* strain HO-1 (Wu et al., 2021), for which the N_2_ produced aerobically is not related to aerobic denitrification, whether the nitrogen removal of *Alcaligenes* strains comes from heterotrophic nitrification and aerobic denitrification (HNAD, the process: NH_4_^+^ → NH_2_OH → NO_2_^–^ → NO_3_^–^ → NO_2_^–^ → NO → N_2_O → N_2_) (Wehrfritz et al., 1993; Richardson et al., 1998, Stein, 2011) or the novel pathway remained to be clarified.

However, it is still not clear (1) whether Dirammox is widely distributed in *Alcaligenes* or not and (2) whether N_2_ generation is exclusively from Dirammox or the combination of Dirammox and HNAD under physiological conditions in *Alcaligenes* strains? In this study, the distribution of *dnfT1RT2ABCD* in *Alcaligenes* genomes was analyzed widely by GenBank. Meanwhile, the sequences of this gene cluster defined in the genomes were further compared and phylogenetically studied. Furthermore, 16 *Alcaligenes* strains were obtained and investigated for their Dirammox and denitrification activities using stable isotope incubations. In addition, the representatives of each phylogenetic type of DnfA were investigated for their enzymatic activity catalyzing oxidation of hydroxylamine to N_2_. The results provided insights into the distribution and evolution of Dirammox in the genus *Alcaligenes*, activity of Dirammox among *Alcaligenes* strains, as well as its mechanism, and thereby the possible importance of Dirammox to nature.

## Materials and Methods

### Bioinformatic Analysis

To investigate the distribution of gene cluster *dnfT1RT2ABCD* in *Alcaligenes* genomes, DnfT1, DnfR, DnfT2, DnfA, DnfB, DnfC, and DnfD of strain HO-1 were used as a query individually against the non-redundant protein sequences database (NR) restricted to *Alcaligenes* genomes to perform BlastP program in NCBI blast web (Johnson et al., 2008), respectively. The algorithm parameter “Max target sequences” was adjusted to 5,000. The posted back results were filtered with conditions, namely, expect threshold ≤0.001, query coverage ≥70%, identity ≥30%, and extract the accession numbers of proteins. Get identical protein groups by the above accession numbers using the NCBI Batch Entrez tool. Then, detailed information about each homologous protein was obtained, including nucleotide accession number, start and stop site in nucleotide, strand, accession number of protein, protein name, organism, strain, and assembly. Finally, only the genomes containing all the seven genes of the *dnf* cluster arranged in one DNA fragment of the strains of a bacterial species were screened as the genomes containing gene cluster *dnfT1RT2ABCD*.

Phylogenetic trees based on 16S rRNA gene sequences or amino acid sequences of DnfAs were reconstructed using the neighbor-joining method (Saitou and Nei, 1987) performed in MEGA X software (Kumar et al., 2018) with 1,000 bootstrap resamplings. Clustal Omega (Sievers and Higgins, 2013) or Clustal W (Thompson et al., 1994) was used to align multiple sequences. ESPript 3.0^1^ (Robert and Gouet, 2014) with default setting was used to render sequence similarities.

### Bacterial Strains, Plasmid, Media, and Cultivation Conditions

Bacterial strains and plasmids used in this study are listed in Table 1. Luria–Bertani medium (LB) was prepared according to Liu Y. et al. (2016). The basal medium was prepared by dissolving KH_2_PO_4_ 0.50 g, Na_2_HPO_4_⋅12H_2_O 1.25 g, MgSO_4_⋅7H_2_O 0.2 g, NaCl 10 g, and trace element solution 2 ml in 1 L of distilled water, pH 7.0–7.5. The trace element solution was prepared according to Joo et al. (2005). A total of 4.72 g of succinate was added as carbon source in the basal medium per liter. Ammonia or nitrite was added as nitrogen source as needed listed in Table 2. *Alcaligenes* strains were cultivated at 30°C, 160 rpm in synthetic media (Table 2). *E. coli* strains were cultivated at 37°C in LB media. Ampicillin (100 mg/L) was supplemented with media as necessary. For agar plates, 1.5% (w/v) of agar was added to the media.

**TABLE 1:** Bacterial strains and plasmids used in this study*.

Strain or plasmid	Description	Source/References
***Alcaligenes* strain**		
*A. ammonioxydans* HO-1	Aerobically converting ^15^NH_4_^+^ to ^15^N_2_ with hydroxylamine accumulation and ^15^NO_2_^–^ to ^15^N_2_O	SHARON bioreactor (Wu et al., 2021)
*A. aquatilis* CGMCC 1.0767	Aerobically converting ^15^NH_4_^+^ to ^15^N_2_ with hydroxylamine accumulation and ^15^NO_2_^–^ to ^15^N_2_O	Rubber
*A. aquatilis* GL12	Aerobically converting ^15^NH_4_^+^ to ^15^N_2_ with hydroxylamine accumulation and ^15^NO_2_^–^ to ^15^N_2_O	Farming wastewater; this study
*A. aquatilis* CGMCC 1.9053	Aerobically converting ^15^NH_4_^+^ to ^15^N_2_ with hydroxylamine accumulation and ^15^NO_2_^–^ to ^15^N_2_O	Activated sludge
*A. faecalis* subsp.*faecalis* PC01	Aerobically converting ^15^NH_4_^+^ to ^15^N_2_ with hydroxylamine accumulation and ^15^NO_2_^–^ to ^15^N_2_O	Farming wastewater; this study
*A. faecalis* subsp. *faecalis* CGMCC 1.1799	Aerobically converting ^15^NO_2_^–^ to ^15^N_2_O	Faecalis (Málek et al., 1963)
*A. faecalis* subsp. *faecalis* CGMCC 1.2006*^T^*	Aerobically converting ^15^NH_4_^+^ to ^15^N_2_ with hydroxylamine accumulation and ^15^NO_2_^–^ to ^15^N_2_O	(Papen et al., 1989)
*A. faecalis* subsp. *faecalis* CGMCC 1.2908	Aerobically converting ^15^NH_4_^+^ to ^15^N_2_ with hydroxylamine accumulation and ^15^NO_2_^–^ to ^15^N_2_O	ND
*A. faecalis* subsp. *faecalis* CGMCC 1.2388	Aerobically converting ^15^NH_4_^+^ to ^15^N_2_ with hydroxylamine accumulation and ^15^NO_2_^–^ to ^15^N_2_O	ND
*A. faecalis* subsp. *faecalis* CGMCC 1.3937	Aerobically converting ^15^NH_4_^+^ to ^15^N_2_ with hydroxylamine accumulation and ^15^NO_2_^–^ to ^15^N_2_O	ND
*A. faecalis* subsp. *faecalis* CGMCC 1.1837	Aerobically converting ^15^NH_4_^+^ to ^15^N_2_ with hydroxylamine accumulation and ^15^NO_2_^–^ to ^15^N_2_O	Tomur Peak, Xinjiang
*A. faecalis* subsp. *phenolicus* DSM 16503*^T^*	Aerobically converting ^15^NH_4_^+^ to ^15^N_2_ with hydroxylamine accumulation and ^15^NO_2_^–^ to ^15^N_2_O	Graywater bioprocessor (Rehfuss and Urban, 2005)
*A. faecalis* subsp. *phenolicus* RL12	Aerobically converting ^15^NO_2_^–^ to ^15^N_2_O and ^15^N_2_	Farming wastewater; this study
*A. faecalis* subsp. *parafaecalis* DSM 13975*^T^*	Aerobically converting ^15^NH_4_^+^ to ^15^N_2_ with hydroxylamine accumulation and ^15^NO_2_^–^ to ^15^N_2_O	Eutrophic garden pond (Schroll et al., 2001)
*A. faecalis* JQ135	Aerobically converting ^15^NH_4_^+^ to ^15^N_2_ with hydroxylamine accumulation and ^15^NO_2_^–^ to ^15^N_2_O	Municipal wastewater (Zhang et al., 2018)
*A.* sp. CGMCC 1.10269	Aerobically converting ^15^NH_4_^+^ to ^15^N_2_ with hydroxylamine accumulation and ^15^NO_2_^–^ to ^15^N_2_O	Dyeing wastewater, this study
***Escherichia coli* strain**		
Transetta(DE3)	Protein expression host	Transgen, China
Transetta(DE3)/pET21a-WP_003803202.1	*E. coli* Transetta(DE3) carrying plasmid pET21a-WP_003803202.1	This study
Transetta(DE3)/pET21a-WP_042487153.1	*E. coli* Transetta(DE3) carrying plasmid pET21a-WP_042487153.1	This study
Transetta(DE3)/pET21a-WP_045930341.1	*E. coli* Transetta(DE3) carrying plasmid pET21a-WP_045930341.1	This study
Transetta(DE3)/pET21a-WP_009459326.1	*E. coli* Transetta(DE3) carrying plasmid pET21a-WP_009459326.1	This study
Transetta(DE3)/pET21a-WP_094197465.1	*E. coli* Transetta(DE3) carrying plasmid pET21a-WP_094197465.1	This study
Transetta(DE3)/pET21a-WP_035272004.1	*E. coli* Transetta(DE3) carrying plasmid pET21a-WP_035272004.1	This study
Transetta(DE3)/pET21a-WP_137431195.1	*E. coli* Transetta(DE3) carrying plasmid pET21a-WP_137431195.1	This study
Transetta(DE3)/pET21a-HCA15598.1	*E. coli* Transetta(DE3) carrying plasmid pET21a-HCA15598.1	This study
**Plasmid**		
pET21a(+)	Amp*^r^*, Expression vactor	Lab stock
pET21a-WP_003803202.1	pET21a(+) haboring *dnfA* of *A. ammonioxydans* HO-1.	This study
pET21a-WP_042487153.1	pET21a(+) haboring *dnfA* of *A. faecalis* subsp. *faecalis* CGMCC 1.2006*^T^*.	This study
pET21a-WP_045930341.1	pET21a(+) haboring *dnfA* of *A. faecalis* subsp. *phenolicus* DSM 16503*^T^*.	This study
pET21a-WP_009459326.1	pET21a(+) haboring *dnfA* (N879_RS10420) of *Alcaligenes* sp. EGD-AK7 (GCA_000465875.3); whole gene synthesis.	This study
pET21a-WP_094197465.1	pET21a(+) haboring *dnfA* of *A. aquatilis* CGMCC 1.9053	This study
pET21a-WP_035272004.1	pET21a(+) haboring *dnfA* of *A. faecalis* subsp. *faecalis* CGMCC 1.3937	This study
pET21a-WP_137431195.1	pET21a(+) haboring *dnfA* (D0C27_RS13980) of *A. faecalis* AU14 (GCA_005311025.1); whole gene synthesis.	This study
pET21a-HCA15598.1	pET21a(+) haboring *dnfA* (DEO64_00430) of *A. faecalis* UBA11281 (GCA_003521065.1); whole gene synthesis.	This study

*CGMCC and DSM denote China General Microbiological Culture Collection Center and German Collection of Microorganisms and Cell Cultures GmbH, respectively. All Alcaligenes strains were tested in a sealed bottle with the V_liquid_:V_bottle_ = 2:25 under 20% O_2_ and 80% He mixed gas.

**TABLE 2:** Nitrogen source used for different media.

Nitrogen source	Medium A	Medium B	Medium C	Medium D	Medium E
5 mM (NH_4_)_2_SO_4_	+	−	−	−	+
5 mM (^15^NH_4_)_2_SO_4_	−	+	+	−	−
3 mM NaNO_2_	−	−	+	+	−
3 mM Na^15^NO_2_	−	−	−	−	+

### Amplification of 16S rRNA Genes and *dnfA*s of Tested Strains

Primers used in this study are listed in Table 3. Primer pair dnfA-upF/dnfA-downR targeting complete *dnfA* were designed based on upstream and downstream sequences of *dnfA*s of *Alcaligenes* fetched from GenBank. High-fidelity DNA polymerase TransStart^®^ FastPfu (Transgen, China) was used. The PCR products were sequenced by a commercial company. Finally, the start and end codons were determined by aligning with *dnfA* sequence of strain HO-1. EMBOSS Transeq^2^ was applied to translate nucleotides into proteins. These sequence data are available in the GenBank^3^ under accession number OM293488-511.

**TABLE 3:** Primers used in this study.

Primer	Sequence (5′→3′)	Description	Source/Reference
27F	AGAGTTTGATCCTGGCTCAG	Universal primers amplifying 16S rRNA gene	Wilson et al., 1990
1492R	GGTTACCTTGTTACGACTT		
dnfA-upF	CAAATCCTTTTAAGCCTGCG	Universal primers amplifying complete *dnfA* of *Alcaligenes*.	This study
dnfA-downR	ACTTTGACGYTTCCGGCCT		
dnfA-F	CGCGGATCATGACWATCAAAAGCTACGAAAC	Universal former primer with *Bam*HI *restriction site* targeting *dnfA* of *Alcaligenes* for clones.	This study
dnfA-R1	CCCAAGCTTTTGCAGCGCCTCCTGTTGTTCG	Reverse primer with *Hin*dIII *restriction site* targeting *dnfA* of *A. faecalis* subsp. *faecalis* CGMCC 1.3937 for cloning pET21a-WP_035272004.1.	This study
dnfA-R2	CCCAAGCTTTTGCAGCGCCTCCTGTTGCTCG	Reverse primer with *Hin*dIII *restriction site* targeting *dnfA* of *A. ammonioxydans* HO-1*^T^* and *A. aquatilis* CGMCC 1.9053, for cloning pET21a-WP_003803202.1 and pET21a-WP_094197465.1, respectively.	This study
dnfA-R3	CCCAAGCTTTTGCAGCGCCTCCTGCTGTTCG	Reverse primer with *Hin*dIII *restriction site* targeting *dnfA* of *A. faecalis* subsp. *faecalis* CGMCC 1.2006*^T^* and *A. faecalis* subsp. *phenolicus* DSM 16503*^T^*, for cloning pET21a-WP_042487153.1 and pET21a-WP_045930341.1, respectively.	This study

### Growth of Ammonia or Nitrite as the Sole Nitrogen Source

Growth (OD_600_) and hydroxylamine were monitored over time when ammonia (medium A, Table 2) was employed as the sole nitrogen source in Erlenmeyer flasks. The ability to grow on nitrite as the sole nitrogen source (medium D, Table 2) was determined over time in sealed bottles as previously described (Wu et al., 2021) but without displacement of the gas of the headspace, in which the seeds were prepared in agar plates, resuspended, and injected into the bottles.

### Nitrogen Removal Characteristics of Dirammox and Aerobic Denitrification

The ability to convert ammonia to N_2_ was tested with ^15^N_2_ production from (^15^NH_4_)_2_SO_4_ (medium B, Table 2) or in the presence of nitrite (medium C, Table 2). For detection of gaseous nitrogen products (N_2_ and N_2_O) of Dirammox and aerobic denitrification, strains were cultivated in media B or E (Table 2) in air-tight bottles prepared according to the previous description (Wu et al., 2021). All samples were assayed when ammonia and formed hydoxylamine were completely consumed.

### Chemicals

^15^N-labeled hydroxylamine (^15^NH_2_OH), Na^15^NO_2_, and (^15^NH_4_)_2_SO_4_ were all 99 atom% ^15^N and got from Cambridge Isotope Laboratories Inc., United States.

### Plasmid Construction, Expression, and Purification of DnfA and the Homologs

In this study, the gene sequences of WP_009459326.1, WP_137431195.1, and HCA15598.1 were obtained from GenBank and synthesized by a commercial company, then the obtained genes were cloned into pET-21a(+) (Table 1). For construction of other plasmids listed in Table 1, PCR products amplified by TransStart^®^ FastPfu were purified and digested with *Bam*HI (NEB) and *Hin*dIII (NEB). The digested amplicons and *Bam*HI/*Hin*dIII digested pET-21a(+) plasmid were ligated by T4 ligase (Takara) at 4°C overnight. For expression of DnfA and its s, *E. coli* Transetta (DE3) containing pET-21a ligating *dnfA* or *dnfAs* from other *Alcaligenes* strains was cultured in LB supplemented with 100 μg/ml ampicillin at 37°C on a rotary shaker (160 rpm) to OD_600_ = 0.3–0.6, then induced with 0.5 mM isopropyl-β-D -thiogalactopyranoside on a rotary shaker (160 rpm) for another 20 h at 16°C. DnfA or its s was purified individually using Ni-NTA resin (QIAGEN) with the following procedures, namely, cells were harvested by centrifugation (5,000 × *g*, 30 min, 4°C), resuspended in buffer A (100 mM Tris-HCl, 100 mM NaCl, 10 mM imidazole, pH 8.0), lysed by ultrasonication, and centrifuged (14,000 × *g*, 30 min, 4°C). Supernatant was applied to Ni-NTA resin (QIAGEN) column pre-equilibrated with buffer A. Ni-NTA matrix was washed with buffer A and added with 10–50 mM imidazole to remove impurities. 6xHis-tagged proteins were eluted with buffer B (100 mM Tris-HCl, 100 mM NaCl, 250 mM imidazole, pH 8.0), desalted with centrifugal filter devices (MilliporeSigma), and excess imidazole was removed with PD-10 desalting column (GE Healthcare).

To analyze the distribution of the genus *Alcaligenes* in nature, BlastN was performed using the 16S rRNA gene sequence (1542 nt) of strain HO-1 as a query against the nucleotide database (NT). Only the sequences with coverage >70% and similarity >95% were included. The isolation source or sample source was extracted and used to analyze the environmental distribution of the genus *Alcaligenes*.

### Hydroxylamine Oxygenase Activity Assays

*In vitro* enzymatic assays, DnfA, and its homologs catalyzing the conversion of hydroxylamine to N_2_ were carried out in 300 μl reaction mixtures in 20 mM buffer Tris-HCl (pH 8.5) containing 330 μM DnfA or its homologs, 10 mM ^15^NH_2_OH, 10 mM NADH, and 20 μM FAD, as described in our preprint^4^ (Wu et al., 2020). Mixtures lacking DnfA were used as the control. The reaction was started with the addition of ^15^NH_2_OH, and the mixture was directly injected into 10 ml gastight tubes whose top air had been completely replaced by 20% O_2_ and 80% He mixed gas. The reactions were incubated at 30°C without agitation in the dark for 100 min. Enzymatic assays were performed in triplicate.

### Analytical Methods and Statistical Analysis

Bacterial growth and concentrations of ammonium (NH_4_^+^) of nitrite (NO_2_^–^), nitrate (NO_3_^–^), hydroxylamine, ^15^N_2_, and ^15^N_2_O were determined as described previously (Wu et al., 2021), except nitrite determine in enzymatic activity system, which was monitored by ion chromatography. For determination of gas products, strain cultivation and DnfA enzymatic reaction were performed in 250 ml sealed bottles containing 20 ml media or 10 ml gastight tube with 300 μl reaction system, respectively. The air was replaced completely by 80% He and 20% O_2_ mixed gas. PerkinElmer Optima 5300EV ICP-OES was used for the determination of atoms, including Fe, Mn, Ca, Cu, K, Mg, Ni, P, Zn, Si, and S. Results are shown as mean ± SD. The correlation relationship was demonstrated by Pearson’s correlation coefficient.

## Results

### Gene Cluster *dnfT1RT2ABCD* Present in the Known Genomes of *Alcaligenes*

**FIGURE 1:**
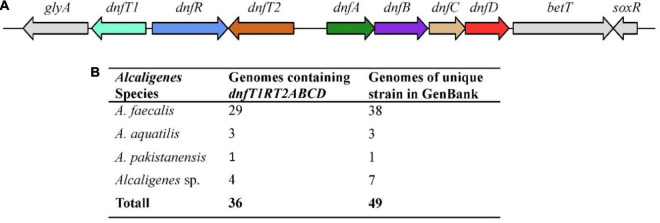
Arrangement **(A)** and statistics on distribution **(B)** of *dnfT1RT2ABCD* among the genomes of the genus *Alcaligenes*. Here, *A. faecalis* includes all three subspecies, namely, *A*. *faecalis* subsp. *faecalis*, *A*. *faecalis* subsp. *Parafaecalis*, and *A*. *faecalis* subsp. *phenolicus*. There are 49 genomes of unique *Alcaligenes* strains in GenBank updated to May 2021, and strain HO-1 was recognized as one of *Alcaligenes* sp.

All the available *Alcaligenes* genomes in GenBank (updated to May 2015) were analyzed for the presence of the gene cluster *dnfT1RT2ABCD*. As shown in Figure 1, a copy (and only one copy) of the gene cluster *dnfT1RT2ABCD* as a whole was defined in the 36 well-assembled *Alcaligenes* genomes (totally 49 genomes of unique *Alcaligenes* strain), including all 19 complete genomes, with the same arrangement and position as those in HO-1 (Figure 1). Among them, *A. faecalis* was the most dominant, with 29 genomes containing *dnfT1RT2ABCD*, followed by unnamed *Alcaligenes* strains (four genomes) and *A. aquatilis* (three genomes) (Figure 1B). In addition, parts of the *dnf* cluster were also defined in the rest of the 15 low-level assembled genomes of the total 49 *Alcaligenes* genomes of unique strain deposited in GenBank. Furthermore, the *dnf* clusters from different *Alcaligenes* genomes exhibited very high sequence similarity (>97% for *dnfABC*), suggesting that the *dnf* cluster was very conserved in the genus *Alcaligenes*. Based on these results, it was deduced that the conserved *dnf* cluster and so the Dirammox were commonly distributed in the genus *Alcaligenes*.

### Aerobic N_2_ Release in *Alcaligenes* Depends on the Activity of Dirammox

**FIGURE 2:**
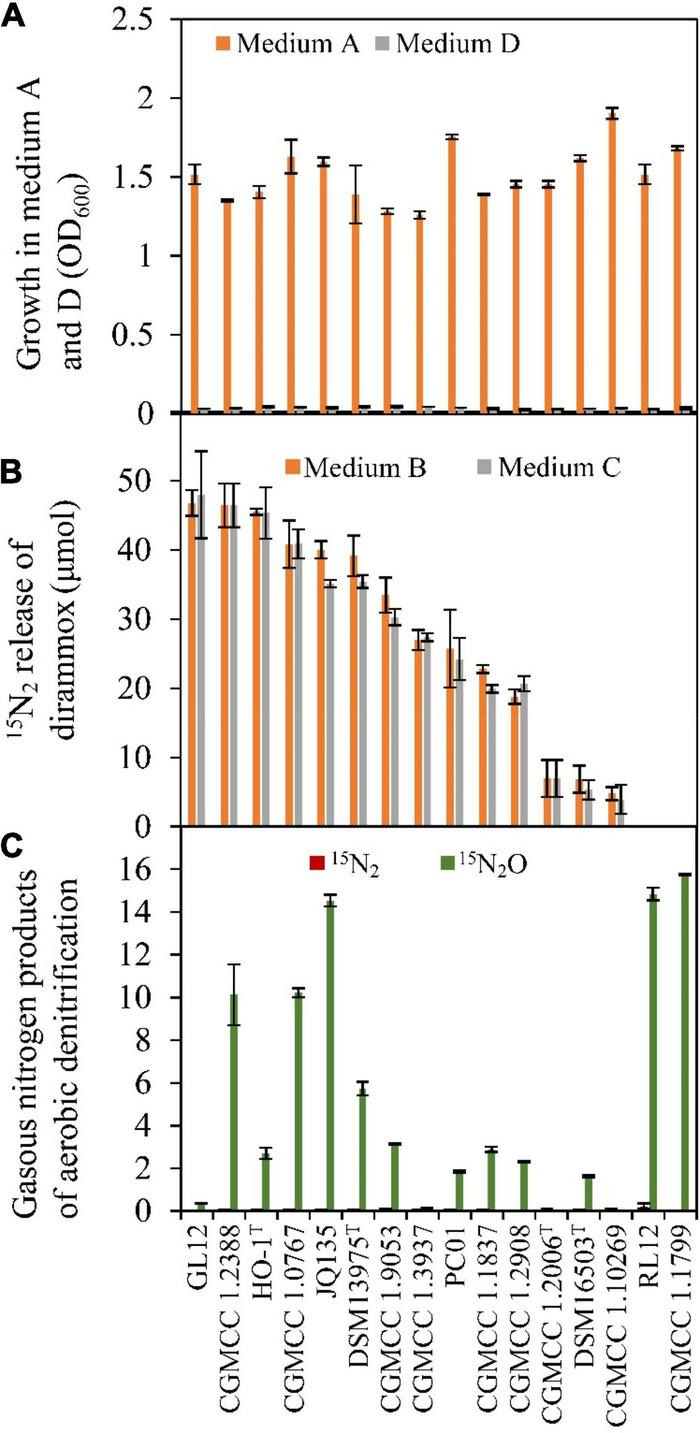
Growth and Dirammox (*dir*ect *amm*onia *ox*idation) activity of *Alcaligenes* strains in different medium. **(A)** Aerobic growth in medium A and medium D, and OD_600_ shown here were data of stationary growth phase. **(B)** ^15^N_2_ release from (^15^NH_4_)_2_SO_4_ (initial amount 100 μmol) in the absence or presence of NaNO_2_ (medium B and medium C, respectively). **(C)** Gaseous products of aerobic denitrification of *Alcaligenes* strain in medium E (60 μmol of initial amount of Na^15^NO_2_).

A total of 16 *Alcaligenes* strains (Table 1) were obtained for verifying the existence of Dirammox pathway and nitrogen removal ability. As shown in Figure 2A, ammonia could be used as the sole nitrogen source for aerobic growth of all *Alcaligenes* strains. In contrast, no growth for all tested strains was observed on nitrite as the sole nitrogen source (Figure 2A), suggesting that *Alcaligenes* spp. have no the ability to assimilate nitrite. Analysis of the gaseous nitrogen products indicated that most of the tested *Alcaligenes* strains (except of strains RL12 and CGMCC 1.1799) could aerobically convert ^15^NH_4_^+^ to ^15^N_2_ when ^15^NH_4_^+^ was used as the sole nitrogen source, in spite of their difference in N_2_-producing ability (Figure 2B). The results in Figure 2B also indicate that ^15^N_2_ amount released was not affected by the addition of nitrite. Figure 2C displays the gaseous nitrogen products released from aerobic denitrification of nitrite by the strains tested. The results showed that no tested strain could produce ^15^N_2_ from nitrite denitrification, and most of them could release ^15^N_2_O from ^15^NO_2_^–^ (medium E, in Table 2) under aerobic conditions. All these results were in good agreement with those of HO-1 (Wu et al., 2021). These results strongly suggested that the production of N_2_ resulted from the direct conversion of ammonia in *Alcaligenes* spp. Therefore, it was concluded that the activity of Dirammox was the only way to produce N_2_ in *Alcaligenes* spp. under aerobic conditions.

### Dirammox Evolves Associated With Members of the Genus *Alcaligenes*

**FIGURE 3:**
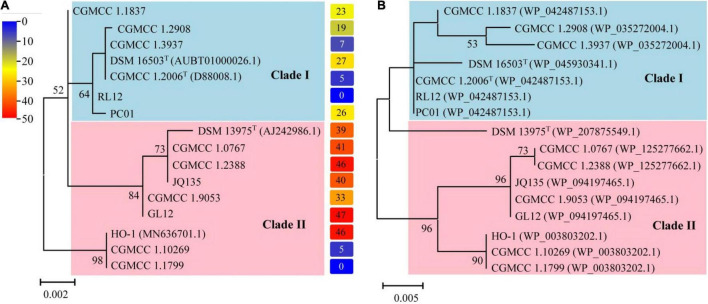
Phylogenetic analyses of the genus *Alcaligenes* based on 16S rRNA gene sequences **(A)** and amino acid sequences of DnfAs **(B)**. The protein accession numbers shown in parentheses represent the best hit of DnfA against NR database (the same in Table 4). The columns on the right of **(A)** represent the heatmap showing the conversion (%) of consumed ^15^NH_4_^+^ to ^15^N_2_ in medium B.

Figure 3 depicts the results of phylogenetic analyses of the genus *Alcaligenes*. It is noticed that the topology of the tree based on 16S rRNA gene sequences (Figure 3A, Table 4) was almost the same as that of the tree based on the amino acid sequences of DnfAs (Figure 3B), and both trees can be divided into two clades, namely, clade I and clade II (Figures 3A,B). Dirammox activities of *Alcaligenes* spp. seemed likely taxon-related, as indicated by the right column of (A) showing the heatmap of conversion (%) of consumed ^15^NH_4_^+^ to ^15^N_2_ in medium B, the members in clade I exhibited lower Dirammox activities than those in clade II with the exception of strains CGMCC 1.10269 and CGMCC 1.1799 (Figure 3), both strains had very low or no Dirammox activities. Furthermore, the G + C contents of *dnf* clusters of *Alcaligenes* spp. were all around 56.3%, a value very close to that (∼57.2%) of the genomes of *Alcaligenes* spp. These results, together with the very high sequence similarity of the *dnf* clusters among *Alcaligenes* species, significantly referred that the *dnf* cluster was evolved associated with the members of the genus *Alcaligenes*. This means that *Alcaligenes* species obtain *dnf* cluster and so the Dirammox genetic potential ability from heredity, but not from horizontal gene transfer from other microbes.

**TABLE 4:** BlastP results of DnfAs against NR database.

Source strain	Best hit accession number	Identity (%)
*A. faecalis* subsp. *phenolicus* DSM 16503*^T^*	WP_045930341.1	100
*A. faecalis* subsp. *phenolicus* RL12	WP_042487153.1	100
*A. faecalis* subsp. *faecalis* CGMCC 1.2006*^T^*	WP_042487153.1	100
*A. faecalis* subsp. *faecalis* PC01	WP_042487153.1	100
*A. faecalis* subsp. *faecalis* CGMCC 1.1837	WP_042487153.1	99.68
*A. faecalis* subsp. *faecalis* CGMCC 1.2908	WP_035272004.1	99.37
*A. faecalis* subsp. *faecalis* CGMCC 1.3937	WP_035272004.1	100
*A. faecalis* subsp. *parafaecalis* DSM 13975*^T^*	WP_207875549.1	100
*A. faecalis* subsp. *faecalis* CGMCC 1.1799	WP_003803202.1	100
*A.* sp. CGMCC 1.10269	WP_003803202.1	100
*A. ammonioxydans* HO-1	WP_003803202.1	100
*A. aquatilis* GL12	WP_094197465.1	100
*A. aquatilis* CGMCC 1.9053	WP_094197465.1	100
*A. faecalis* JQ135	WP_094197465.1	100
*A. aquatilis* CGMCC 1.0767	WP_125277662.1	100
*A. faecalis* subsp. *faecalis* CGMCC 1.2388	WP_125277662.1	100

### *In vitro* Activities of DnfA Homologs Catalyzing the Direct Oxidation of Hydroxylamine to N_2_

**FIGURE 4:**
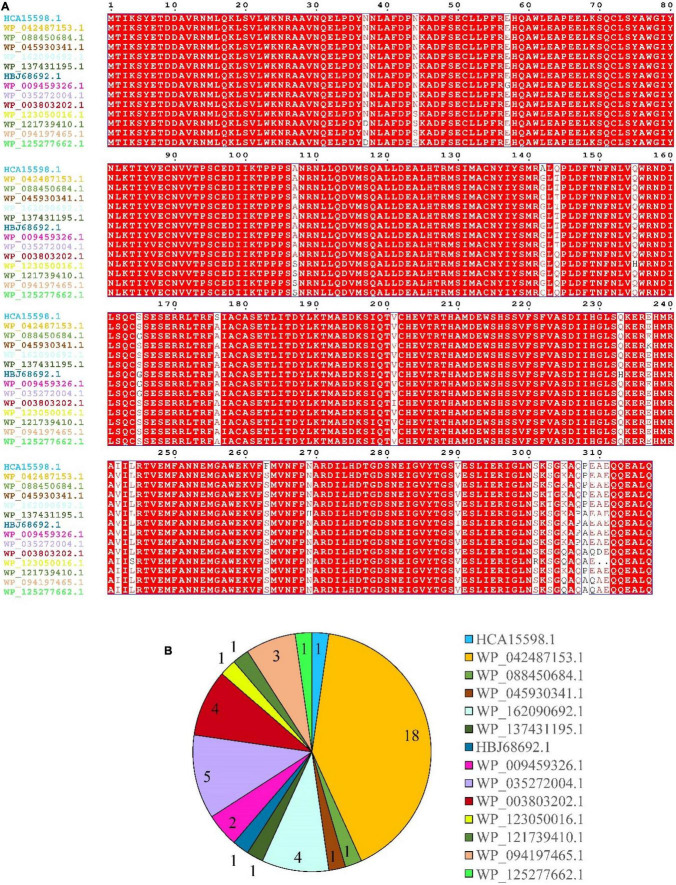
Multiple-sequence alignment **(A)** and statistics **(B)** of *Alcaligenes* DnfAs. Accession numbers of the proteins (DnfAs, or DnfA homologs) are shown. All genomes (49) in Figure 1 were analyzed, and five of them with incomplete DnfA sequence were excluded.

Results of multiple sequence alignment of DnfA and its homologs (DnfAs) from *Alcaligenes* species showed only a few amino acid differences (Figure 4A), suggesting that the *Alcaligenes* DnfAs share the same function. In addition, Figure 4A also shows that *Alcaligenes* DnfAs can be divided into 14 subtypes. Among them, DnfA with accession number WP_042487153.1 was the most abundant one that existed in 18 genomes, followed by DnfA with accession number WP_035272004.1, which existed in five genomes (Figure 4B). To verify the functions of DnfAs, some representatives were selected and their genes were obtained by PCR amplification or gene synthesis for their enzymatic activity assays.

**FIGURE 5:**
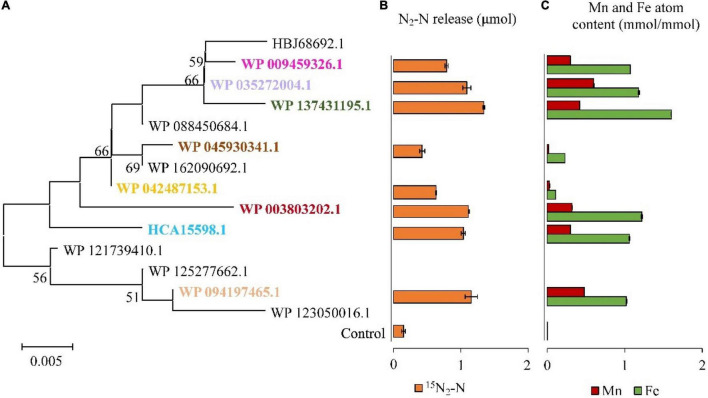
Hydroxylamine oxidation activities of DnfAs from *Alcaligenes* strains. Phylogenetic tree of the representatives of all DnfA homologs defined in the genomes of all *Alcaligenes* strains deposited in GenBank **(A)**; ^15^N_2_ release of the representatives of DnfA homologs from ^15^NH_2_OH under 20% O_2_ and 80% He *in vitro* **(B)**. The content of Fe and Mn atoms of DnfAs **(C)**. The data are shown as the mole content of metal atoms per mole of DnfA.

It was found that DnfAs could catalyze the direct oxidation of hydroxylamine to N_2_ with a molecular ratio of 2:1 in the presence of molecular O_2_, FAD, and NADH. Therefore, *in vitro* reconstitution enzymatic activity assays using FAD as the chemical electron mediator were performed for DnfAs as shown in Figure 5, according to previous method (Choi et al., 2008). The results (Figure 5B) indicated that all the enzyme reaction samples could release ^15^N_2_ from ^15^NH_2_OH, in spite of the different amounts of ^15^N_2_ released, suggesting that all tested DnfAs could oxidize hydroxylamine directly to N_2_. These results also showed that the different original DnfAs exhibited different enzyme activity. DnfAs with accession numbers WP_042487153.1 and WP_045930341.1 released 0.63 ± 0.01 and 0.43 ± 0.04 μmol ^15^N_2_-N, respectively, showing the lowest activity (Figure 5B). The results also suggested that the *in vitro* enzymatic activities of the tested DnfAs seemed likely none phylogenetically related, a phenomenon quite different from the Dirammox activities of *Alcaligenes* strains (Figure 3). Furthermore, when combined with the results presented in Figure 3, an interesting fact was noticed. The *Alcaligenes* strains with low *in vitro* DnfA activity also exhibited low Dirammox activity, such as strains RL12, CGMCC 1.2006*^T^*, PC01, and DSM 16503*^T^*. In contrast, the strains with low Dirammox activity may harbor DnfA of high *in vitro* activity, such as strain CGMCC 1.3937.

Based on homology alignment against the public database, DnfA was annotated as a diiron oxygenase and related to the AurF *N*-oxygenase (Choi et al., 2008; Leipoldt et al., 2017) and CmlIs (Knoot et al., 2016; Komor et al., 2018). Similarly, DnfAs are predicted to contain a diiron motif. In this case, atoms of DnfAs were determined. Only Fe and Mn existed in DnfA, and the results (Figure 5C) showed that all the tested DnfAs contained both Fe and Mn atoms with contents ranging from 0.11 ± 0.00 to 1.60 ± 0.01 and from 0.03 ± 0.00 to 0.48 ± 0.00 mmol per mmol of protein, respectively (Figure 5C). Notably, a positive correlationship was defined between the *in vitro* activities (release amount of ^15^N_2_) of DnfAs and the contents of Fe, Mn, or Fe + Mn with correlation coefficient *r* = 0.92, 0.86, and 0.93, respectively.

### Distribution of the Genus *Alcaligenes* in Environments

The above results indicated that Dirammox pathway was commonly distributed in the genus *Alcaligenes*, in spite of a few *Alcaligenes* species exhibiting low or even no ability of transforming ammonia to N_2_ under 20% oxygen. In this case, the distribution of the genus *Alcaligenes* in environments was investigated first based on 16S rRNA gene sequences.

**FIGURE 6:**
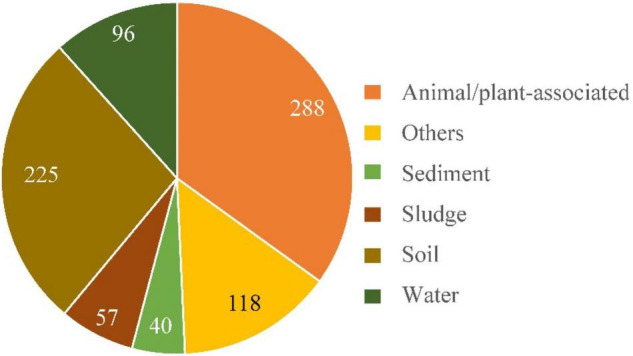
Distribution of the genus *Alcaligenes* in nature. Categories of samples and numbers of *Alcaligenes* 16S rRNA gene sequences are indicated. The results were based on the analysis of 16S rRNA gene sequences defined in GenBank of NCBI database.

For concise purpose, samples were briefly divided into five categories, namely, soil, water, sediment, sludge, and animal–plant-associated, according to their sources. Each category includes samples from both natural environments and human-made surroundings. Clinical samples and those directly derived from animals or plants were classified as animal/plant-associated samples. A total of 824 different *Alcaligenes* 16S rRNA gene sequences with definite environment/source information were defined in NT database of the GenBank. The results (Figure 6) showed that there were more *Alcaligenes* 16S rRNA gene sequences (∼35%, 288/824) from animal/plant-associated samples than other samples, followed by soil samples (27.3%, 225/824). Also, there were 11.7, 6.90, and 4.85% (96, 57, and 40 of 824, respectively) of the *Alcaligenes* 16S RNA gene sequences from samples of water, sludge, and sediment, respectively. These results indicated that members of the genus *Alcaligenes* are widely distributed in various environments with quite high biodiversity, in good agreement with the results described in the literature as mentioned in the “Introduction” section 1. Therefore, the genus *Alcaligenes* is an important conductor of elemental biogeochemical cycles, including the nitrogen cycle, and so the Dirammox pathway is probably important to the nitrogen cycle since most members of the genus *Alcaligenes* could exhibit Dirammox activity (Figure 2B). In addition, approximately 31% (256/824) *Alcaligenes* strains were derived from WWTPs or various industrial effluents and agricultural wastewater. This means that *Alcaligenes* might also be efficient candidates for wastewater treatment.

## Discussion

In our previous work, a novel microbial N_2_ production pathway, termed Dirammox, was defined in *Alcaligenes* strain HO-1 and encoded by the gene cluster *dnfT1RT2ABCD* (Wu et al., 2021). Recently, *dnfABC* was further proved to be the genetic basis for Dirammox and regulated by *dnfR* in another *Alcaligenes* strain, JQ135 (Xu et al., 2022). Here, it was found that high homogeneity of Dirammox was among the members of the genus *Alcaligenes*. Our results showed that the production of N_2_ from ammonia by an *Alcaligenes* strain must be the results of Dirammox pathway, but not the results of aerobic denitrification that could only emit N_2_O (Figure 2) under aerobic conditions. The results are quite different from previous studies on nitrogen removal of *Alcaligenes* strains (van Niel et al., 1992; Joo et al., 2006; Liu et al., 2015; Shoda and Ishikawa, 2016; Chen et al., 2021), which all attributed to N_2_ producing or nitrogen removal to HNAD. Although it was also speculated that N_2_ generation in *Alcaligenes* strains was through hydroxylamine (NH_4_^+^ → NH_2_OH → N_2_O → N_2_) (Joo et al., 2006; Zhao et al., 2012), the pathway and molecular basis remained unclear. Here, we proved the use of Dirammox, a process of direct oxidization of ammonia to N_2_ *via* hydroxylamine in *Alcaligenes* strains.

In this study, this gene cluster *dnfT1RT2ABCD* as a whole was investigated for its distribution in members of the genus *Alcaligenes*, and it was found that *dnf* cluster not only existed in all members of the genus *Alcaligenes* (Figure 1), but also was highly conserved among *Alcaligenes* species. Phylogenetic data revealed that DnfA was taxon-related (Figure 3), referring to the *Alcaligenes* species acquiring Dirammox pathway by heredity but not horizontal gene transfer from other microbes, which in turn determines different Dirammox activities among *Alcaligenes* members in different clades. The function and activity of DnfA are of course important, but other conditions, such as low or even no transcription or expression of *dnf* genes and the activity of DnfB or DnfC, will also influence the Dirammox activity. So, we can see strains CGMCC 1.10269 and CGMCC 1.1799 breaking the rule, whose Dirammox activities differ from their very closely related neighbors HO-1 (Figure 3).

DnfA, annotated as a diiron oxygenase, contained both Fe and Mn atoms whose content greatly affected the *in vitro* activities of DnfA catalyzing the direct oxidation of hydroxylamine to N_2_. It was reported that Mn and Fe have similar atomic numbers and Fe*^II^*/Fe*^III^* and Mn*^II^*/Mn*^III^* are common active states in many proteins. Although DnfAs were predicted to contain a diiron site, Mn atoms might take up the site in place of Fe atoms. This means that the contents of Fe or Mn or Fe + Mn were a very important factor determining the *in vitro* activities or even physiological activities of DnfAs, and this needs further investigation in the future. The differences of Fe and Mn atoms among *Alcaligenes* spp. might result from the sequences of amino acid of DnfA, and the change of some amino acid residues might decrease the combination of protein and Fe and Mn. The reasons for this might be quite complex and need to be further revealed in the future.

With general N_2_-producing ability of *Alcaligenes* members and the wide distribution of the genus *Alcaligenes* in nature as shown above, it was speculated that Dirammox was also important to the nitrogen cycle. Certainly, the Dirammox activity *in situ* and the assessment of N-fluxes contributed by Dirammox conducted by *Alcaligenes* or even other bacteria should be investigated urgently in the near future.

## Conclusion

In this study, the cluster *dnfT1RT2ABCD* encoding Dirammox proved to be universally distributed, conserved, and dependently evolved in the genus *Alcaligenes*. Most of the *Alcaligenes* strains exhibited Dirammox activities, which seemed likely taxon-related, in spite of their different performance. In contrast, the *in vitro* activities of DnfAs catalyzing the direct oxidation of hydroxylamine to N_2_ were not taxon-related but depended on the contents of Fe and Mn ions. Dirammox might be important to the nitrogen cycle, as well as the environment, since the genus *Alcaligenes* was widely distributed in environment.

## Data Availability Statement

The datasets presented in this study can be found in online repositories. The names of the repository/repositories and accession number(s) can be found in the article/supplementary material.

## Author Contributions

Z-PL and S-JL contributed to conception. L-LM and T-TH designed the study, analyzed data, and wrote the first draft of the manuscript. T-TH and LM contributed to the data collection. Z-PL reviewed and edited the manuscript. J-SP, QH, YL, and M-RW provided resources for the study. G-MA built the N_2_ and N_2_O detection methods. All authors contributed to manuscript revision, read and approved the submitted version.

## Conflict of Interest

The authors declare that the research was conducted in the absence of any commercial or financial relationships that could be construed as a potential conflict of interest.

## Publisher’s Note

All claims expressed in this article are solely those of the authors and do not necessarily represent those of their affiliated organizations, or those of the publisher, the editors and the reviewers. Any product that may be evaluated in this article, or claim that may be made by its manufacturer, is not guaranteed or endorsed by the publisher.

## Footnotes

1
http://espript.ibcp.fr

2
https://www.ebi.ac.uk/Tools/st/emboss_transeq/

3
www.ncbi.nlm.nih.gov

4
https://doi.org/10.1101/2020.08.20.256677
